# Pressure Mat Analysis of Walk and Trot Gait Characteristics in 66 Normal Small, Medium, Large, and Giant Breed Dogs

**DOI:** 10.3389/fvets.2018.00256

**Published:** 2018-10-16

**Authors:** Maria A. Fahie, Jonathan C. Cortez, Marc Ledesma, Yuhua Su

**Affiliations:** ^1^College of Veterinary Medicine, Western University of Health Sciences, Pomona, CA, United States; ^2^VCA Yorba Regional Animal Hospital, Anaheim, CA, United States; ^3^The Ohio State University, Columbus, OH, United States; ^4^Dr. Su Statistics, Kaunakakai, HI, United States

**Keywords:** pressure mat, gait characteristics, dog, size, breed

## Abstract

**Objectives:** To document temporospatial variables and gait symmetry measured by the GAITRite® system for normal, healthy dogs at the walk and trot with the leash side recorded.

**Study Design:** Observational, prospective, cohort study.

**Sample Population:** 66 healthy dogs of various common breeds with no evidence of lameness that were small (< 10 kg), medium (10- < 25 kg), large (25- < 40 kg), or giant (≥40 kg).

**Methods:** Dogs walked and trotted at their preferred velocity on a pressure sensing walkway system. Video observation confirmed inclusion criteria were met for three valid trials at each gait for each dog. Coefficients of variance were used to summarize the data for analysis. Fore and hindlimb ratios were compared. Gait symmetry was assessed with the leash on the left and right side.

**Results:** Coefficients of variation for gait parameters ranged from 20 to 28% for all except velocity and hind reach. There was no statistically significant difference in differences in fore and hindlimb ratios for stance %, GLS, TPI, or step:stride ratio, across weight categories or between walk and trot. Less than 8% of normal dogs had a GLS score < 90 (indicating lameness). Leash side did influence gait symmetry, since GLS, TPI, and step:stride all had statistically significant differences in means between leash side, irrelevant of the weight category or gait.

**Conclusions and Clinical Relevance:** This system allowed simple, reliable gait assessment and values reported may be considered normal reference ranges for temporospatial variables collected with this system within the weight ranges and gaits reported. Controlling leash side and patient size is recommended for therapeutic intervention studies.

## Introduction

Quantitative gait analysis systems have become an invaluable tool in monitoring gait while comparing procedures and treatments (1). Pressure walkway systems used to measure temporospatial variables (TSV) have been shown to be simple and efficient in obtaining multiple gait cycles with little variability (2–10). Several published reports use such systems for objective assessment of gait in response to therapeutic interventions (11–15).

Established inclusion criteria for clinical studies are vital to achieving consistent, statistically significant results, however there is not a universally accepted method. The potential influence of controlling dog velocity during gait analysis is not resolved. Studies using the same pressure walkway system as this one controlled velocity by using a metronome the handler matched as they walked (16), or by setting inclusion criteria to a certain velocity range (5) or by simply allowing the dog gait velocity preference (6). Leash side may influence dogs to shift their weight away from the leash as reported in 5 small dogs (16). Breed conformation may also affect results (5).

The issue of calibration of pressure walkway systems was recently published (17). The system used in that and other studies assumes to calculate a force due to the pressure that is exerted on the paw (2, 4, 9, 17). The system used in the present study is initially calibrated at the manufacturer and since it does not use force as one of the measurements, repeated calibration is not necessary prior to each use. Parameters generated by the various walkway systems are quite different and cannot be compared for clinical research.

The purpose of this study is to document the parameters measured by the GAITRite® system for normal, healthy dogs of the various weight categories (< 10 kg, 10- < 25 kg, 25- < 40 kg, ≥40 kg) at walk and trot. We hypothesized there might be differences in TSVs or gait symmetry among the dog sizes or based on leash side.

## Materials and methods

### Equipment

The walkway system (GAIT4 Dog® walkway, CIR Systems Inc., Sparta, NJ) used in this study was identical to prior reports (5, 16). The system consisted of a 5.8 × 0.6 m portable mat with 18,432 encapsulated sensors. The active dimensions of the mat were 4.9 × 0.6 m. A 1.25 × 0.85 m section of inactive mat was placed at each end of the walkway system to provide a transitional entrance and exit. The mat was calibrated by the manufacturer before purchase as previously described.^5^ Digital video recording of each pass was made using a camera positioned at one end of the walkway system and used for visual gait assessment, scoring of the passes and footfall verification.

### Inclusion criteria

Healthy, adult, client-owned dogs of various breeds were enrolled in the study after approval by our institutional animal care and use committee. The dogs were acclimated to the boarding facility and accustomed to being walked on a leash. They all had normal body condition score (4–6/9) as determined visually by applying American Animal Hospital Association guidelines, and no history or presence of detectable orthopedic or neurologic abnormalities. No animals with a pacing gait were included. Dogs were grouped into categories by weight, defined as small (<10 kg), medium (10- <25 kg), large (25- <40 kg), and giant (≥40 kg). Dogs were measured according to previously published reports (5, 16) and walked by a trained handler (JC) with left or right leash side recorded. Walks were scored on a scale of 0–5 with 0 being a perfect pass with no head motion or leash pulling, 1 being a very slight leash pull at some point during the pass, 2 being a slight leash pull or slight head motion at the beginning or end of the walk, 3 being much leash pulling and head turning, 4 being stopping on the mat during the pass, and 5 being the disastrous walk where those patient motions are constant. Only walks scored 0–2 were included in the study.

### Data processing

Videos of each pass were reviewed by one author (MF) to ensure inclusion criteria were met. Three walk and three trot passes were selected for each dog with leash side recorded. The software program (GAITFour software version 4.9Wr, CIR Systems Inc., Sparta, NJ, United States) was used to determine measured parameters. Parameters analyzed included velocity, stance %, GAIT4 Dog® Lameness Score (GLS), Hind Reach (HR), Total Pressure Index (TPI), step:stride ratio and number of sensors activated. Velocity is obtained by dividing distance traveled by ambulation time and is expressed as centimeters per second. Stance percent is the percentage of stance time, the weight bearing portion of each gait cycle, compared to stride time, the time elapsed between the first contacts of two consecutive footfalls of the same foot. GLS is calculated considering weight distribution, based on observed to expected TPI by limb and established body type loading ratios (default 60:40) and should be approximately 100%. Hind Reach (HR) was measured along the line of progression, from the heel center of the hind paw to the heel center of the previous fore paw on the same side. A negative value of HR could result if the dog fails to bring the heel point of the hind paw forward of the previous fore paw. Total pressure index (TPI) was the sum of peak pressure values recorded from each activated sensor by a paw during mat contact. Expected TPI values were about 30 for each fore paw and 20 for each hind paw. Step:stride ratio was used to assess gait symmetry and was expected to be about 50% if the dog's gait was symmetric. The number of sensors activated was dependent on how much of the paw activates the sensors, independent of gait velocity. The overall coefficient of variation for stance %, GLS, TPI and step: stride ratio were calculated by dividing each SD by the mean, multiplying each by 100, then adding those values and dividing by the number of dogs.

### Statistical analysis

Several analyses were conducted using SAS 9.4 (SAS Institute Inc., Cary, NC) in order to achieve the goals of this study. The ratio between forelimbs (LF:RF) and the ratio between hindlimbs (LH:RH) of stance %, GLS, TPI, and step:stride were computed. Differences in the two ratios were then calculated for the forelimb parameters vs. the hindlimb parameters in different sized dogs, between walk and trot, and between handler and leash positions (left or right). Linear mixed-effects models (18, 19) were conducted for each parameter. No random effects were constructed. The compound symmetry covariance structure was used to model the dependence between observations for each dog. The predictors considered in the model include weight category (small, medium, large, giant), walk vs. trot, and handler (right vs. left). The F test was used to test if the effect of a predictor was statistically significant. A *p* < 0.05 indicated significance. Estimated marginal means were computed for the significant effects. Note that estimated marginal means (mean response for each factor, adjusted for any other variables in the model) are not the same as the arithmetic means (mean response for each factor, not adjusted for any other variables in the model). Quantile-quantile (QQ) plots of the scaled residuals (obtained after multiplying the raw residuals by Cholesky decomposition) (18, 20) were used to assess the multivariate normality assumption of the linear mixed-effects models. To further investigate Hind Reach, a 2-sample *t*-test was used to determine if there was a statistically significant difference in hind reach between limbs with no lameness (normal GLS of 100) vs. those with lameness (abnormal GLS 10% different from 100). The Cochran-Mantel-Haenszel (CMH) test (21) was used to determine if there was a relationship between limb and lameness, after controlling for walk/trot. Chi-square tests of independence were performed to determine if there was an association between lameness and limb, within each group of dogs (walk vs. trot). The CMH test was also used to determine if there was a relationship between weight category and step:stride ratio within 95% confidence, after controlling for limb. To determine if one gait (walk or trot) provided more consistent results in certain parameters (velocity, stance %, GLS, TPI, and step:stride) compared to walk, means, standard deviations and coefficient of variation (CV) were computed.

## Results

Eighty-three dogs were walked, 66 dogs were chosen based on the inclusion criteria. The 17 dogs excluded from the study did not have enough valid passes or data recorded to satisfy study inclusion criteria. There were 11 small (<10 kg), 25 medium (10–25 kg), 20 large (25–40 kg), and 10 giant (>40 kg) dogs. Small dogs included chihuahuas, small terriers, dachshund and mixed breeds. Medium dogs included medium terriers, schnauzers, spaniels, French bulldogs, and mixed breeds. Large dogs included boxers, pointers, bulldogs, large retrievers and mixed breeds. Giant dogs included Newfoundland, Great Dane, giant retrievers, and mixed breeds.

### Symmetry

Tables 1, 2 summarize the mean ± SD for velocity, stance %, GLS, HR, TPI, step:stride for the 4 weight categories. Table 3 documents overall coefficients of variation for velocity, stance%, GLS, HR, TPI, and step:stride at walk and trot. There was no statistically significant difference in differences in fore and hindlimb ratios for stance %, GLS, TPI, or step:stride ratio, across weight categories or between walk and trot. However, there was a statistically significant difference in differences in fore and hindlimb ratios for GLS, TPI, and step:stride ratio between the handler and leash on the right vs. the left side documented in Table 4.

**Table 1:** Data at the walk expressed as mean ± standard deviation.

	** < 10 kg L**	** < 10 kg R**	**10- < 25 kg L**	**10- < 25 kg R**	**25- < 40 kg L**	**25- < 40 kg R**	**>40 L**	**>40R**
	***n* = 7**	***n* = 26**	***n* = 26**	***n* = 49**	***n* = 19**	***n* = 41**	***n* = 11**	***n* = 19**
Velocity(cm/s)	75.3 ± 31.0	111.8 ± 55.2	124.4 ± 55.3	147.5 ± 70.9	131.3 ± 66.5	164.4 ± 66.7	107.2 ± 47.9	134.0 ± 65.4
Stance%LF	51.5 ± 21.3	72.1 ± 28.3	55.9 ± 15.7	51.8 ± 11.9	56.4 ± 14.2	56.0 ± 8.6	56.0 ± 19.9	58.4 ± 14.2
Stance%RF	52.2 ± 21.6	50.4 ± 11.5	53.7 ± 12.0	51.2 ± 11.8	55.4 ± 14.7	55.8 ± 8.6	56.6 ± 20.2	58.9 ± 14.0
Stance%LH	46.2 ± 20.1	42.9 ± 12.8	49.6 ± 12.1	47.4 ± 12.3	53.0 ± 14.2	52.7 ± 10.0	55.1 ± 19.6	54.4 ± 15.4
Stance%RH	45.4 ± 19.8	43.1 ± 12.2	49.4 ± 12.1	47.1 ± 12.1	53.0 ± 14.1	52.7 ± 9.9	55.1 ± 19.7	54.9 ± 14.9
GLS LF	89.3 ± 36.5	101.8 ± 16.9	89.9 ± 23.7	100.0 ± 12.0	96.6 ± 20.6	98.5 ± 6.0	82.9 ± 29.2	92.7 ± 19.2
GLS RF	90.8 ± 36.9	97.6 ± 16.6	98.9 ± 18.0	99.8 ± 12.3	95.3 ± 20.0	94.2 ± 5.7	87.5 ± 31.1	91.8 ± 19.1
GLS LH	79.8 ± 32.8	96.4 ± 17.9	94.6 ± 17.6	98.3 ± 13.5	95.4 ± 20.7	102.1 ± 9.0	94.5 ± 33.6	101.7 ± 21.4
GLS RH	87.9 ± 35.9	94.0 ± 17.5	96.5 ± 19.2	96.5 ± 12.4	97.4 ± 21.1	103.2 ± 8.7	100.8 ± 36.4	103.0 ± 21.5
HR LH	3.1 ± 8.8	0.8 ± 5.9	5.1 ± 7.4	5.0 ± 7.0	13.1 ± 11.4	14.3 ± 9.0	15.1 ± 33.6	15.1 ± 11.4
HR RH	2.7 ± 9.0	1.0 ± 5.8	5.5 ± 7.5	5.5 ± 7.6	13.2 ± 11.3	14.0 ± 8.6	15.9 ± 14.3	16.1 ± 11.6
TPI% LF	26.8 ± 10.9	30.6 ± 5.1	29.4 ± 5.3	30.0 ± 3.6	29.0 ± 6.2	29.5 ± 1.8	24.8 ± 8.8	27.8 ± 5.8
TPI% RF	27.3 ± 11.1	29.1 ± 5.2	29.6 ± 5.4	29.9 ± 3.7	28.6 ± 6.0	29.4 ± 1.7	26.2 ± 9.3	31.8 ± 23.3
TPI% LH	15.9 ± 6.5	19.3 ± 3.6	18.9 ± 3.5	19.7 ± 2.7	19.1 ± 4.2	20.4 ± 1.8	18.8 ± 6.7	20.3 ± 4.3
TPI% RH	17.6 ± 7.2	18.8 ± 3.5	19.3 ± 3.8	19.3 ± 2.5	19.5 ± 4.2	20.7 ± 1.7	19.7 ± 7.6	20.6 ± 4.3
Step:stride LF	43.3 ± 17.5	49.5 ± 7.7	44.7 ± 15.9	47.6 ± 10.4	36.9 ± 22.6	48.1 ± 10.3	44.3 ± 15.7	57.7 ± 40.0
Step:stride RF	44.1 ± 17.8	48.4 ± 7.3	48.3 ± 8.4	49.4 ± 5.6	47.8 ± 9.8	49.5 ± 1.5	45.5 ± 16.1	47.9 ± 9.8
Step:stride LH	44.0 ± 17.8	52.8 ± 7.4	51.2 ± 8.5	48.7 ± 5.7	48.1 ± 10.0	50.1 ± 4.1	44.0 ± 15.6	46.9 ± 9.9
Step:stride RH	43.3 ± 17.5	49.2 ± 8.1	48.6 ± 8.5	50.0 ± 5.8	48.0 ± 10.0	48.8 ± 3.8	45.7 ± 16.2	49.1 ± 10.4

*L, Handler and leash on left; R, Handler and leash on right; n, number of data points; kg, kilograms; LF, left front; RF, right front; LH, left hind; RH, right hind; GLS, GAIT4Dog® Lameness Score; HR, hind reach; TPI, total pressure index*.

**Table 2:** Data at the trot expressed as mean ± standard deviation.

	**<10 kg L**	**<10 kg R**	**10- <25 kg L**	**10- <25 kg R;**	**25- <40 kg L**	**25- <40 kg R;**	**>40 L**	**>40R**
	***n* = 18**	***n* = 16**	***n* = 36**	***n* = 36**	***n* = 15**	***n* = 39**	***n* = 8**	***n* = 12**
Velocity(cm/s)	127.7 ± 60.5	133.8 ± 65.4	165.1 ± 68.8	169.4 ± 65.8	228.8 ± 73.3	179.7 ± 70.3	209.2 ± 112.0	185.3 ± 73.9
Stance%LF	45.9 ± 13.4	45.1 ± 13.0	48.3 ± 12.3	48.1 ± 13.0	43.2 ± 12.3	51.5 ± 11.4	36.5 ± 19.3	51.2 ± 13.3
Stance%RF	45.6 ± 12.9	45.5 ± 12.3	47.5 ± 11.8	47.2 ± 11.8	4.50 ± 14.7	51.4 ± 11.8	36.0 ± 19.1	52.1 ± 13.3
Stance%LH	39.2 ± 12.7	36.0 ± 12.3	43.6 ± 12.9	42.0 ± 12.2	38.4 ± 11.5	47.4 ± 12.7	31.7 ± 16.9	47.2 ± 13.9
Stance%RH	48.9 ± 27.6	36.5 ± 12.5	43.0 ± 12.5	42.0 ± 12.2	38.1 ± 11.2	47.9 ± 12.6	32.5 ± 17.3	47.1 ± 13.5
GLS LF	102.1 ± 20.9	95.4 ± 23.3	100.6 ± 15.4	100.7 ± 15.3	93.8 ± 25.5	97.2 ± 12.8	77.5 ± 40.9	92.3 ± 19.7
GLS RF	99.3 ± 204	96.3 ± 23.3	99.5 ± 15.3	100.3 ± 15.7	95.8 ± 27.0	97.3 ± 13.0	77.2 ± 40.7	92.6 ± 19.7
GLS LH	91.3 ± 19.7	94.5 ± 23.8	95.7 ± 16.6	95.2 ± 17.8	94.6 ± 26.8	99.5 ± 14.7	83.6 ± 44.3	100.1 ± 21.7
GLS RH	88.4 ± 20.2	95.0 ± 24.0	94.9 ± 15.3	94.1 ± 16.3	90.6 ± 27.0	102.0 ± 15.2	85.1 ± 45.3	102.3 ± 22.5
HR LH	−2.1 ± 4.9	0.8 ± 6.3	3.4 ± 7.2	3.0 ± 5.5	8.0 ± 8.4	10.4 ± 9.9	4.4 ± 5.0	8.7 ± 12.9
HR RH	−1.7 ± 5.1	0.6 ± 6.1	3.6 ± 7.7	3.5 ± 5.9	7.1 ± 9.3	10.2 ± 9.6	5.3 ± 5.3	9.5 ± 13.7
TPI% LF	30.7 ± 6.3	28.6 ± 7.0	30.2 ± 4.6	30.2 ± 4.6	28.0 ± 7.6	29.2 ± 3.9	23.2 ± 12.2	27.7 ± 5.9
TPI% RF	29.9 ± 6.1	28.9 ± 7.0	29.8 ± 4.6	30.1 ± 4.7	28.7 ± 8.1	29.2 ± 3.9	23.1 ± 12.2	27.8 ± 5.9
TPI% LH	18.2 ± 3.9	18.9 ± 4.8	19.1 ± 3.3	19.0 ± 3.6	18.9 ± 5.4	19.9 ± 3.0	16.8 ± 8.9	20.0 ± 4.3
TPI% RH	17.7 ± 4.1	19.0 ± 4.8	19.0 ± 3.1	18.8 ± 3.3	18.1 ± 5.4	20.4 ± 3.0	17.0 ± 9.0	20.5 ± 4.5
Step:stride LF	48.8 ± 10.1	47.7 ± 10.7	49.2 ± 7.1	46.3 ± 13.1	47.3 ± 12.7	45.5 ± 14.9	41.1 ± 21.7	48.3 ± 10.1
Step:stride RF	47.9 ± 9.6	47.5 ± 10.7	53.8 ± 33.9	52.8 ± 35.5	46.2 ± 12.4	48.9 ± 6.0	38.8 ± 20.4	47.5 ± 10.0
Step:stride LH	47.6 ± 9.4	48.1 ± 10.8	49.4 ± 7.1	48.4 ± 6.9	52.9 ± 39.1	49.8 ± 6.3	40.4 ± 21.3	47.5 ± 10.1
Step:stride RH	49.2 ± 10.6	47.3 ± 10.6	48.5 ± 7.0	49.6 ± 7.1	44.0 ± 13.4	48.5 ± 6.2	39.4 ± 20.8	48.3 ± 10.3

*L, Handler and leash on left; R, Handler and leash on right; n, number of data points; kg, kilograms; LF, left front; RF, right front; LH, left hind; RH, right hind; GLS, GAIT4Dog® Lameness Score; HR, hind reach; TPI, total pressure index*.

**Table 3:** Overall Coefficients of Variation (CV) at walk and trot.

**Measure**	**Gait**	**CV %**
Velocity (cm/s)	Walk	40.9
	Trot	37.9
Stance %	Walk	25.6
	Trot	28.6
GLS	Walk	20.4
	Trot	23.9
Hind Reach	Walk	173.9
	Trot	169.9
TPI %	Walk	21.0
	Trot	21.8
Step:stride	Walk	24.8
	Trot	25.7

*GLS, GAIT4Dog® Lameness Score; TPI, Total Pressure Index*.

**Table 4:** Results of the mixed models.

**Measure**	**Effect**	**DF1**	**DF2**	**F**	***p*-value**
Stance %	Weight	3	63	0.25	0.85
	Walk/trot	1	61	0.18	0.67
	Leash side	1	57	0.22	0.63
GLS	Weight category	3	63	0.32	0.81
	Walk/trot	1	61	0.25	0.61
	Leash side	1	57	6.37	0.01*
TPI	Weight category	3	63	0.44	0.72
	Walk/trot	1	61	0.61	0.43
	Leash side	1	57	6.12	0.01*
Step:stride	Weight	3	63	1.11	0.35
	Walk/trot	1	61	2.76	0.10
	Leash side	1	57	6.91	0.01*

*DF1, Numerator degrees of freedom for the F-statistic; DF2, Denominator degrees of freedom for the F-statistic; GLS, GAIT4Dog® Lameness Score; TPI, Total Pressure Index*.

* *indicates significance at the 0.05 level*.

### Velocity

CV for velocity were consistent for walked and trotted dogs of any size.

### Gait4 dog® lameness score

In general, GLS scores should be about 100. The observation of gait by video assessment of the board-certified surgeon (MF) and handler (JC) did not reveal any signs of lameness. Despite that, of the 1528 observations of GLS scores, 122 (7.98%) were considered “lame” (GLS <90). For walk, there was no association between limb and lameness (*p* = 0.1103). The percentage of lame limb was very close for each limb, 6% for LF, 7% for LH, 6% for RF, and 11% for RH. For trot, there was an association between limb and lameness (*p* <0.0001). The percentage of lame limb was higher for the hindlimbs (16% for LH and 14% for RH) than for the fore limbs (2% for LF and 3% for RF).

### Hind reach

Mean HR for both hindlimbs was in general larger for walk than for trot across the 4 weight groups. Estimated marginal mean differences in HR between walk and trot were 1.80 (small), 5.86 (medium), 12.55 (large), and 15.28 (giant), with a statistically significant difference in HR between walk and trot in medium, large, and giant (all *p* <0.0001) dogs but not small dogs. The difference in HR between walk and trot increased with the size of the dogs.

## Discussion

The present study reports normal gait characteristics across a variety of common breeds within multiple weight categories applicable to clinical patients. To the authors' knowledge, there are no other studies using this pressure walkway system reporting this variety of dog size and gait. Consistent data was able to be collected in healthy dogs of all sizes.

### Symmetry

The degree of variation of gait symmetry considered normal has yet to be determined (21) but in the present study, symmetry was confirmed with the step:stride ratio values since 87–93% of values were within the 95% confidence interval of the expected value of 50. In general, TPI is expected to be about 30/30/20/20 for the LF/RF/LH/RH limbs, respectively. The fore and hindlimb ratios for stance %, GLS, TPI and step:stride were not significantly different for any size dog at the walk or trot. Leash side did influence gait symmetry, since GLS, TPI, and step:stride all had statistically significant differences in means between leash side, irrelevant of the weight category or gait.

### Influence of gait velocity

In the present study, dogs were allowed velocity preference and both walk and trot were assessed for consistency. The variation in velocity was not consistently better for all sizes of dogs at either gait. Small breeds maintained a more consistent velocity at the walk, while giant breeds were more consistent trotting. Medium and large breeds were equally consistent at walk and trot. Since data collection is simple with this system, the most informative data for comparing interventions would include both gaits when testing small and giant breeds.

### GAIT4 dog® lameness score (GLS)

GLS is a unique parameter for this system software and to the author's knowledge is not reported prior. The author's subjective visual assessment of dog videos did not reveal lameness but almost 8% of them received a GLS score < 90 on one limb, suggesting lameness. It is possible that the system was detecting subtle lameness in those dogs, which is one of its clinically applicable features. The variation could also have been related to the score of the pass, with a higher score indicating some head motion or slight leash pull during the pass, or leash side, if the dog was shifting weight away from the handler, as previously reported. (16) Dogs may also have a dominant forelimb, behavioral lateralization, or paw preference as reported by Schneider. (22) In that study, the Kong (KONG Company, Golden, CO) paw preference test (23) documented 63% of dogs demonstrated preferential paw usage, with about 34% left-pawed, 28% right-pawed, and 37% ambilateral. To the authors' knowledge, the potential effect of paw preference on gait analysis has not been tested, although if a patient is compared to itself in interventional studies, the effect should be inconsequential.

### Hind reach

The present study documented much variation in hind reach in all weight categories, although at walk the CV was lower than any other parameter. Various breeds have vastly different conformation and this parameter is dependent upon leg length and length of the body. Smaller dogs with shorter legs may have negative values for hind reach if they do not bring the heel point of their hind paw forward of their previous ipsilateral forepaw. Differences in fore and hindlimb muscling among breeds is likely also a contributing factor. Hind reach (HR) was accurate in the few limbs deemed lame by the GLS score, in that the HR was shorter than the contralateral hindlimb if the GLS score was < 90. HR was also larger for walk than trot across all weight groups, which may be pertinent from the point of view of both postoperative rehabilitation monitoring and therapeutic intervention studies. Further studies of hind reach in specific breeds of dogs may elucidate patterns associated with certain conditions causing lameness.

### Comparison of data with prior published reports

There are 3 prior published reports of normal dogs that include parameter details for comparison to this study. The first detailed published study (5) of this system included Labrador retrievers weighing 17.7–35.5 kg. The present study weight categories fall between those numbers, although the symmetry ratios were similar to those reported in the present study for the two weight categories that fall into that weight range. The CVs for parameters in the present study were lower than a prior report (16) that included five small dogs and various handlers. Perhaps our increased sample size and consistent handler explain the variance reduction. Specifically, our TPI means were similar, however the hind reach means were quite different (present study: LH 1.65, RH 2.09, reference 15: −5.75 and −5.44, respectively). Kim et al. (4), used a different pressure walkway system, but also compared normal small and large dogs, and concluded that the mean stance phase duration of the hindlimbs was significantly shorter than the forelimbs in small dogs (4). Our results are similar, with a greater difference in stance % between fore and hindlimbs in small dogs compared to medium, large and giant dogs. However, data of this present study did not indicate overall TSV differences in the various sizes of dogs as the prior study concluded (4). The present study TPI was about LF 30, RF 30, LH 20, RH 20 for all sizes of dogs at both walk and trot. This differed from those of Carr et al. (10), who identified gait differences between Border Collies and Labrador Retrievers presumed related to their intended working purpose. It is also different from anticipated in a prior study (5) which suggested larger dogs with larger paws would activate more sensors and exert a greater TPI. The number of sensors activated in this study did increase with dog size but did not influence anticipated TPI values.

## Study limitations

Limitations of the present study include materials and methods details. A disadvantage intrinsic to use of a pressure sensing walkway and temporospatial gait analysis system includes the inability to measure forces in three dimensions, and thus only being able to quantitate a product of total ground reaction force. Theoretically, dogs could be exerting craniocaudal forces that would not be detected from a simple vertical pressure analysis, which could affect their limb kinematics. Data generated in this study are unique to this system. There are inherent problems with subjective gait assessment performed live and with video, however at least this study included only one observer for consistency, although increasing chances for bias. No dogs had radiographic evaluation to determine whether there was any underlying orthopedic disease. Dogs with GLS scores <90 were not further assessed with examination or radiographs to confirm a problem. This study grouped the dogs by weight, rather than breed, and since hind reach and possibly other parameters are affected by leg length compared to body length, results may have been affected by the inclusion of chondrodystrophic and brachycephalic breeds. The sample size for leash side data analysis was balanced with more values taken with the leash on the right side (238) compared with the left (140). Dogs well-trained to their handler on a certain side, more commonly the left, were more difficult to obtain low scoring, perfect walk/trot passes. This may have weakened the power of the statistical analysis.

## Conclusions and clinical relevance

This system allowed simple, reliable gait assessment. We recommend controlling leash side and patient size for therapeutic intervention studies. The values presented in this study may be considered normal reference ranges for temporospatial variables (TSV) from this system within the weight ranges and gaits reported.

## Ethics statement

This study was carried out in accordance with the recommendations of the Western University of Health Sciences, College of Veterinary Medicine, Institutional Animal Care, and Use committee with written informed consent from all subjects. The approval number was R11/IACUC/009.

## Author contributions

MF mentored JC and ML, who were veterinary students at the time of the data collection, in authoring the Morris Animal Foundation grant. They performed the data collection and wrote first drafts of the manuscript. Since their graduation MF has done all manuscript authoring/editing. YS did all of the statistical analysis and authored those sections of the manuscript.

### Conflict of interest statement

The authors declare that the research was conducted in the absence of any commercial or financial relationships that could be construed as a potential conflict of interest.

## Abbreviations

CV: coefficient of variation
GLS: GAIT4 Dog® Lameness Score
HR: hind reach
LF: left front
LH: left hind
LL: leash on the left
LR: leash on the right
RF: right front
RH: right hind
TPI: total pressure index
TSV: temporospatial variables.
